# Comparative review of *Francisella tularensis* and *Francisella novicida*

**DOI:** 10.3389/fcimb.2014.00035

**Published:** 2014-03-13

**Authors:** Luke C. Kingry, Jeannine M. Petersen

**Affiliations:** Division of Vector-Borne Diseases, Bacterial Diseases Branch, Centers for Disease Control and PreventionFort Collins, CO, USA

**Keywords:** tularemia, *Francisella tularensis*, *Francisella novicida*, intracellular pathogen, virulence

## Abstract

*Francisella tularensis* is the causative agent of the acute disease tularemia. Due to its extreme infectivity and ability to cause disease upon inhalation, *F. tularensis* has been classified as a biothreat agent. Two subspecies of *F. tularensis, tularensis* and *holarctica*, are responsible for tularemia in humans. In comparison, the closely related species *F. novicida* very rarely causes human illness and cases that do occur are associated with patients who are immune compromised or have other underlying health problems. Virulence between *F. tularensis* and *F. novicida* also differs in laboratory animals. Despite this varying capacity to cause disease, the two species share ~97% nucleotide identity, with *F. novicida* commonly used as a laboratory surrogate for *F. tularensis*. As the *F. novicida* U112 strain is exempt from U.S. select agent regulations, research studies can be carried out in non-registered laboratories lacking specialized containment facilities required for work with virulent *F. tularensis* strains. This review is designed to highlight phenotypic (clinical, ecological, virulence, and pathogenic) and genomic differences between *F. tularensis* and *F. novicida* that warrant maintaining *F. novicida* and *F. tularensis* as separate species. Standardized nomenclature for *F. novicida* is critical for accurate interpretation of experimental results, limiting clinical confusion between *F. novicida* and *F. tularensis* and ensuring treatment efficacy studies utilize virulent *F. tularensis* strains.

## Introduction and overview

*F. tularensis* was first isolated in 1912 as the causative agent of a plague-like disease affecting squirrels in Tulare county, California (McCoy and Chapin, 1912). Subsequently, Edward Francis, for whom the genus is named, established that several clinical syndromes in humans were caused by *F. tularensis* and proposed the name “tularemia” to describe the illness (Francis, 1925). In 1950 researchers isolated a bacterium from salt water collected from Ogden Bay, Great Salt Lake, Utah (Larson et al., 1955)**. Initial experiments revealed the bacterium resembled *F. tularensis* morphologically, but fermented sucrose, was of lesser virulence than *F. tularensis* and did not cross-react with serum from rabbits inoculated with heat, ether, formalin, or phenol killed *F. tularensis* (Larson et al., 1955). Based on these phenotypic differences, the isolate was given the unique species name *F. novicida* (Larson et al., 1955)**. The classification of *F. novicida* as a distinct species was further substantiated in 1964 as a result of its less fastidious growth requirements as compared to *F. tularensis* and the lack of heterologous vaccine protection in mice immunized with killed *F. novicida* and challenged with several *F. tularensis* strains (Owen et al., 1964). Additionally, guinea pigs injected subcutaneously with rabbit immune serum against either *F. tularensis* or *F. novicida* followed by challenge with antigen preparations from both *F. tularensis* and *F. novicida* showed antibody adsorption to homologous but not heterologous antigen at the sight of serum injections (i.e., passive cutaneous anaphylaxis assay) (Owen et al., 1964).

DNA-DNA hybridization experiments performed with *F. tularensis* and *F. novicida* in the 1980s indicated a high degree of genetic relatedness between the two (≥92% at 50°C; ≥86% at 65°C) (Hollis et al., 1989). On this basis, it was proposed, though not validly published that *F. novicida* be reassigned as a subspecies of *F. tularensis* (*F. tularensis* subsp. *novicida*) (Hollis et al., 1989; Lapage, 1992; Tindall et al., 2006). To meet Bacteriological Code requirements, a proposal to transfer *F. novicida* to the subspecies rank of *F. tularensis* (i.e., *F. tularensis* subsp. *novicida*) was validly published in 2010 in the International Journal of Systematic and Evolutionary Microbiology (IJSEM) (Tindall et al., 2006; Huber et al., 2010). This proposal was met with formal objection in IJSEM, as it was based solely on genetic relatedness and did not take into consideration the documented phenotypic and genomic differences between *F. tularensis* and *F. novicida* (Johansson et al., 2010). Indeed, in the 2010 publication proposing reclassification of *F. novicida*, 11 metabolic traits unique to *F. novicida* as compared to *F. tularensis* were identified but not considered with respect to its suggested reassignment as *F. tularensis* subsp. *novicida* (Huber et al., 2010).

In recent decades, an explosion in the amount of basic research focused on understanding and treating tularemia has occurred due to concern about the intentional misuse of *F. tularensis* as a bioweapon (Dennis et al., 2001; Cowley and Elkins, 2011). Research studies using virulent *F. tularensis* strains can be particularly challenging, as they can only be handled under BSL-3 conditions and, in the U.S., within laboratories that are Tier 1 select agent approved (Federal Register, 2012). In contrast, the *F. novicida* type strain U112 is exempt from select agent regulations in the U.S. and can be handled under standard BSL-2 laboratory conditions (Federal Register, 2012). Information about select agent exemptions for *F. tularensis* can be found at the following website www.selectagents.gov/Select%20Agents%20and%20Toxins%20Exclusions.html#francisella. The less stringent containment requirement for *F. novicida* U112, its high genetic identity to *F. tularensis*, its ability to infect macrophages *in vitro*, to cause illness in laboratory mice, and ease of genetic manipulation as compared to *F. tularensis* have all contributed to widespread use of *F. novicida* U112 as a surrogate for *F. tularensis* (Anthony et al., 1991; Mdluli et al., 1994; Schmerk et al., 2009; Cowley and Elkins, 2011).

To date, the appropriate nomenclature for *F. novicida* remains controversial and non-standardized. While *F. novicida* is recognized on the Approved List of Bacterial Names (Skerman et al., 1980), *F. tularensis* subsp. *novicida* is validly published (Huber et al., 2010), resulting in two different names and no clear decision on the correct nomenclature. As a result, a variety of names including, but not limited to, *F. tularensis, F. tularensis* subsp. *novicida, Ft novicida, Ftn, Ftt, Fn*, and *F. novicida*, have all been used in the published literature. This lack of standardized terminology is further complicated by non-enforcement of consistent nomenclature by journals and editors. It is particularly problematic when trying to interpret published experimental results obtained using *F. novicida* U112, but described only as *F. tularensis* with no strain information included. Another negative outcome of the proposed classification of *F. novicida* as a subspecies of *F. tularensis* is that other *F. novicida* strains, excluding the exempt U112 strain, are considered select agents in the US, despite the fact they do not cause tularemia. In order to support maintaining separate species designations for *F. novicida* and *F. tularensis*, genomic as well as clinical, virulence, ecologic and pathogenic differences between the two organisms are reviewed here (Table 1). We also discuss the utility of *F. novicida* as a laboratory surrogate for *F. tularensis* with respect to treatment of tularemia.

**Table 1:** **Genetic and phenotypic differences between *F. tularensis* and *F. novicida***.

		***F. tularensis*^a^**	***F. novicida*^b^**	**References**
Genome	Size	1,892,819 bp	1,910,031 bp	Larsson et al., 2005; Rohmer et al., 2007
	Protein coding genes	1445	1731	Larsson et al., 2005; Rohmer et al., 2007
	Pseudogenes	254	14	Rohmer et al., 2007
	FPI	2 copies	1 copy	Nano et al., 2004; Larsson et al., 2005
	Restriction modification systems	1 gene	4 functional systems/6 genes	Gallagher et al., 2008
	CRISPR/Cas	No	Yes	Sampson et al., 2013; Schunder et al., 2013
	O-antigen	15 genes	12 genes; aa identity to Ft 98% to 20%	Thomas et al., 2007; Sjödin et al., 2012
	Recombination	No	Yes	Larsson et al., 2009
	IS element proliferation	Yes	No	Larsson et al., 2009
Clinical	Tularemia	Yes	No	Francis, 1925
	Transmission	Vector-borne, animal contact, inhalation of aerosols	Salt water; brackish water	Larson et al., 1955; Dennis et al., 2001; Brett et al., 2012; Whitehouse et al., 2012
Ecology	Animal hosts	Zoonotic: small mammals, lagomorphs	No	Hopla, 1974; Jellison, 1974
	Arthropod hosts	Ticks, flies, mosquitoes	No	Jellison, 1974; Petersen et al., 2009b
Virulence (LD_50_ by subcutaneous or intradermal route of infection)	Mice	1 CFU	Range from 10 to >10^7^ CFU	Bell et al., 1955; Larson et al., 1955; Olsufiev et al., 1959; Owen et al., 1964; Meshcheriakova et al., 1995; Kieffer et al., 2003
	Guinea pig	1 CFU	Range from 10 to >10^5^ CFU	Bell et al., 1955; Olsufiev et al., 1959; Meshcheriakova et al., 1995
	Rabbit	1 CFU	>10^8^ CFU	Olsufiev et al., 1959; Meshcheriakova et al., 1995
(LD_50_ by intranasal or intratracheal route of infection)	Mice	<10 CFU	Approximate LD_50_ of 10 CFU	Lauriano et al., 2004; Pechous et al., 2008
	Fischer 344 rats	5 × 10^2^ CFU	Approximate LD_50_ of 5 × 10^6^ CFU	Ray et al., 2010
Mechanisms of pathogenicity	Cytokine induction upon cellular uptake	No	Yes	Butchar et al., 2008; Dai et al., 2013
	Inflammasome activation	Delayed	Yes	Mariathasan et al., 2006; Weiss et al., 2007b; Fernandes-Alnemri et al., 2010; Dotson et al., 2013
	CRISPR/cas mediated TLR2 evasion	No	Yes	Dai et al., 2013; Sampson and Weiss, 2013a,b
	PI3K/Akt signaling	No; miR-155 suppressed	Yes; miR-155 induced	Cremer et al., 2009
	Pulmonary cell association	Alveolar MΦ /dendritic cells	Alveolar MΦ /neutrophils	Hall et al., 2008
	O-antigen role	Intracellular replication	Complement resistance	Thomas et al., 2007; Case et al., 2014
	Knockout of *iclR* or *galE* genes attenuates virulence in mice	No	Yes	Mortensen et al., 2010; Thomas et al., 2011

a *Specific numbers given are in reference to F. tularensis subsp. tularensis Schu S4, except for virulence which is from (Olsufiev et al., 1959) strain Schu*.

b *Specific numbers given are in reference to F. novicida U112*.

## Human disease and transmission

*F. tularensis* is one of the most infectious bacterial pathogens known. Studies in human volunteers in the 1960s demonstrated that infection was established with as few as 25 organisms when aerogenically exposed to *F. tularensis* subsp. *tularensis*, with clinically overt disease occurring 3–5 days post exposure (McCrumb, 1961). Two subspecies of *F. tularensis, tularensis* (also called type A) and *holarctica* (also called type B), cause human tularemia (Petersen and Molins, 2010). Between these two subspecies, disease outcome and geographic distribution differs (Olsufiev et al., 1959). *F. tularensis* subsp. *tularensis* causes disease only in North America and is associated with higher mortality in humans as compared to *F. tularensis* subsp*. holarctica*, which causes less severe illness throughout the Northern Hemisphere (Olsufiev et al., 1959). Human infection due to a third subspecies, *F. tularensis* subsp. *mediasiatica*, has never been documented in the published literature.

Within both *F. tularensis* subsp. *tularensis* and subsp. *holarctica*, distinct subpopulations have been delineated by a number of different genotyping methods (Petersen and Molins, 2010). In the case of *F. tularensis* subsp. *tularensis*, pulsed field gel electrophoresis defined three subpopulations, A1a, A1b, and A2, which differ with respect to clinical outcome (Kugeler et al., 2009). Among patients infected with A1b strains, significantly higher fatality rates were observed as compared to those patients infected with A1a or A2 strains (Kugeler et al., 2009). The higher mortality rate for infection with an A1b strain was not associated with host factors (age, sex, underlying illness), indicating an intrinsic characteristic of A1b strains (i.e., virulence) is responsible for the observed difference (Kugeler et al., 2009).

*F. tularensis* causes the zoonotic, vector-borne disease tularemia. Clinical expression of tularemia in humans depends primarily on the route of transmission (Tärnvik and Berglund, 2003; WHO, 2007). Humans acquire infections by a variety of different mechanisms, including arthropod bites (ticks, flies, mosquitoes), direct contact with infected animals (e.g., skinning animals after hunting), ingestion of water or food contaminated by infected animals, and inhalation of infective aerosols (Dennis et al., 2001; WHO, 2007). For all forms, fever and acute symptoms are hallmarks of tularemia in healthy individuals. Arthropod transmission of *F. tularensis* causes glandular and ulceroglandular forms of tularemia, with the latter form of disease presenting as an ulcer at the site of the arthropod bite. Skinning infected animals also leads to ulceroglandular tularemia. Other forms of tularemia include oculoglandular tularemia, acquired via direct inoculation of the eye; oropharyngeal tularemia, acquired through ingestion of water or food contaminated by infected animals; and pneumonic (respiratory) tularemia, acquired through inhalation of infective aerosols during landscaping, farming, or laboratory activities. It is the pneumonic form of tularemia that is the most severe and of highest concern with respect to an intentional aerosol event (Dennis et al., 2001).

In comparison to *F. tularensis, F. novicida* infection is not associated with healthy individuals. *F. novicida* infection in humans is exceedingly rare and therefore often difficult to diagnose accurately (Brett et al., 2012; Birdsell et al., 2009). Only 12 cases have been documented (Hollis et al., 1989; Clarridge et al., 1996; Leelaporn et al., 2008; Birdsell et al., 2009; Brett et al., 2012; Respicio-Kingry et al., 2012; Sjödin et al., 2012; Whitehouse et al., 2012). An *F. novicida*-like infection was reported in an Australian patient, however, genome comparisons indicate the strain is more similar to *F. hispanensis* (Whipp et al., 2003; Sjödin et al., 2012). Illness caused by *F. novicida* does not resemble tularemia. Clinical information available for 11 reported cases indicate that 9 of the *F. novicida* cases occurred in patients who were immuno-compromised or had underlying health problems (Hollis et al., 1989; Clarridge et al., 1996; Leelaporn et al., 2008; Birdsell et al., 2009; Brett et al., 2012; Respicio-Kingry et al., 2012; Whitehouse et al., 2012). Fever and acute disease, hallmarks of tularemia in healthy individuals, were only observed for *F. novicida* infections in compromised patients (Hollis et al., 1989; Clarridge et al., 1996; Leelaporn et al., 2008; Brett et al., 2012; Respicio-Kingry et al., 2012; Whitehouse et al., 2012). In the two healthy individuals with *F. novicida* infection, regional lymphadenopathy, lacking fever or other symptoms, was reported (Hollis et al., 1989; Birdsell et al., 2009). Classic forms of tularemia, including ulceroglandular, pneumonic, oropharyngeal, and oculoglandular, have not been observed for *F. novicida* infection in healthy individuals.

Given the rarity of *F. novicida* infection in humans, little is known with regards to how the organism is transmitted. Reported human infections are associated with uncertain routes of exposure. For those cases where the mode of infection was ascertained, two cases were due to near-drowning events in salt water and three cases were associated with environmental contamination of outdoor ice machines (Brett et al., 2012; Respicio-Kingry et al., 2012; Whitehouse et al., 2012). No evidence exists to suggest that *F. novicida* is transmitted by animals or arthropod vectors (see Ecology section).

## Ecology

In nature, *F. tularensis* and *F. novicida* occupy distinct ecological niches; *F. tularensis* is a classic vector-borne zoonotic pathogen, whereas *F. novicida* is not. As an intracellular pathogen, *F. tularensis* (both subsp. *tularensis* and subsp. *holarctica*) infects and causes disease and mortality in a large number of animal hosts (Hopla, 1974; Jellison, 1974). The bacterium is most often associated with lagomorphs and rodents, including voles, squirrels, and beavers. *F. tularensis* is also found in nature in a number of arthropod vectors, including ticks, flies, and mosquitoes, which bite both animal and human hosts and thereby transmit the organism (Petersen et al., 2009b). Maintenance of *F. tularensis* in nature involves a cycle in which mammals serve as the amplifying hosts and arthropod vectors feed on these bacteremic hosts to disseminate the bacterium to other animals (Petersen et al., 2009b).

In contrast to *F. tularensis*, the identification of *F. novicida* has never been reported in wild animals (healthy or moribund), indicating that in nature *F. novicida* is not a zoonotic bacterium. *F. novicida* has also never been identified in arthropod vectors in nature. Moreover, in the case of arthropods, the lack of identification of *F. novicida* does not appear to be due to inadequate testing methods as numerous *Francisella*-like endosymbionts have been identified in ticks via PCR and sequencing (Scoles, 2004; Goethert and Telford, 2005; Kugeler et al., 2005; Machado-Ferreira et al., 2009; De Carvalho et al., 2011; Ivanov et al., 2011; Kreizinger et al., 2013). As arthropod vectors characteristically acquire infection from bacteremic animal hosts, the lack of identification of *F. novicida* in arthropods is consistent with the presumed inability *F. novicida* to cause bacteremia in wild animals. It is likely that *F. novicida* resides in an environmental niche and is propagated in nature via a mechanism that does not involve mammalian or arthropod hosts. Indeed, the sole source of *F. novicida* isolates to date has been salt water. This includes the *F. novicida* U112 type strain as well as 9 other *F. novicida* isolates (Larson et al., 1955; Petersen et al., 2009a; Whitehouse et al., 2012). Additional environmental sources of *F. novicida*, including brackish water and soil, have been implicated based on PCR detection and sequencing analysis (Barns et al., 2005; Kuske et al., 2006; Berrada and Telford, 2010).

## Genomics

Genome sequencing has been performed on several *F. tularensis* and *F. novicida* strains, with a limited number of genomes fully assembled and annotated (Larsson et al., 2005; Beckstrom-Sternberg et al., 2007; Chaudhuri et al., 2007; Rohmer et al., 2007; Barabote et al., 2009; Champion et al., 2009; Larsson et al., 2009; Modise et al., 2012; Sjödin et al., 2012; Svensson et al., 2012; Antwerpen et al., 2013). Consistent with the high degree of genetic similarity previously determined via DNA-DNA re-association (Hollis et al., 1989), the average nucleotide identity observed across 1.1 MB of genomic sequence from 3 *F. novicida* strains and 13 *F. tularensis* strains (8 subsp. *holarctica*, 1 subsp. *mediasiatica*, 2 subsp. *tularensis* A1, and 2 subsp. *tularensis* A2) is ≥97.7% (Larsson et al., 2009). Despite this high degree of nucleotide identity, differences are apparent between their respective genomes. *F. novicida* U112 has a larger genome of 1,910,031 bases with more protein coding genes (1731) as compared to *F. tularensis*. The genome size of *F. tularensis* subsp *holarctica* LVS and *F. tularensis* subsp. *tularensis* Schu S4 is 1,895,998 and 1,892,819 bases, with 1380 and 1145 protein coding genes, respectively (Rohmer et al., 2007; Larsson et al., 2009).

Selective genome reduction in the intracellular pathogen *F. tularensis* is clear; the *F. tularensis* subsp. *tularensis* Schu S4 and *F. tularensis* subsp. *holarctica* LVS genomes contain 254 and 303 pseudogenes, respectively (Rohmer et al., 2007). In contrast, only 14 pseudogenes are evident in the *F. novicida* U112 genome (Rohmer et al., 2007). Larsson et al. identified a total of 279 gene losses present in six *F. tularensis* genomes (3 subsp. *holarctica*, 1 subsp. *mediasiatica*, and 2 subsp. *tularensis* strains) as compared to the *F. novicida* U112 genome (Larsson et al., 2009). Frequently it is components of metabolic pathways that are deleted during the transition to an intracellular pathogen, as the nutrients can be acquired from the host. Indeed, metabolic differences between *F. novicida* and *F. tularensis* date back to early characterization of *F. novicida*, when it was found to be less fastidious compared to *F. tularensis* (Owen et al., 1964). More recently, Huber *et al*. identified 11 different metabolic traits present only in *F. novicida* as compared to *F. tularensis* subsp. *tularensis, F. tularensis* subsp. *holarctica*, and *F. tularensis* subsp. *mediasiatica* (Huber et al., 2010). Genomic analyses of *F. novicida* U112, *F. tularensis* subsp. *tularensis* Schu S4, and *F. tularensis* subsp. *holarctica* LVS indicate 41.2 percent of the genes predicted to be involved in amino acid biosynthesis in *F. novicida* U112 are inactivated in one or both *F. tularensis* strains (Rohmer et al., 2007). *F. novicida* U112 appears to have 3 incomplete amino acid synthesis pathways (lysine, histidine, and methionine) whereas in *F. tularensis* subsp. *tularensis* Schu S4 there are 9 incomplete pathways (arginine, histidine, lysine, tyrosine, methionine, cysteine, threonine, valine, and isoleucine) (Larsson et al., 2005; Rohmer et al., 2007; Meibom and Charbit, 2010; KEGG, 2014).

The *F. novicida* U112 genome encodes 84 genes (including those involved in amino acid biosynthesis) that are inactivated in both *F. tularensis* subsp. *tularensis* Schu S4 and *F. tularensis* subsp. *holarctica* LVS (Rohmer et al., 2007). The predicted function of these genes (carbohydrate metabolism, amino acid biosynthesis, metabolite transport, energy metabolism, transport, and DNA restriction/modification) is consistent with *F. novicida* maintaining the ability to exist in the environment, outside animal hosts. For example, *F. novicida* U112 encodes 4 intact restriction barrier systems in its genome that impair acquisition of foreign methylated DNA by as much as 10^6^ fold over native *F. novicida* U112 DNA, suggesting *F. novicida* resides in a niche where it encounters foreign DNA (Maier et al., 2004; Gallagher et al., 2008). The majority of genes encoding restriction barrier systems in *F. tularensis* genomes (subsp. *tularensis* Schu S4 and WY96-3418, subsp. *holarctica* LVS, FTA, and OSU18, and subsp. *mediasiatica*) are present in the form of pseudogenes, suggesting that with its transition to an intracellular pathogen, restriction barrier systems were no longer necessary for survival (Gallagher et al., 2008). This evolutionary phenomenon is also present in strains of increasing virulence in both *Yersinia* and *Burkholderia* (Ong et al., 2004; Kim et al., 2005; Gallagher et al., 2008). Another example of *F. novicida* retaining functions for environmental survival and persistence is the identification of 5 genes (FTN_0451-0456) encoded in the *F. novicida* U112 genome that are responsible for the synthesis and breakdown of the secondary messenger, bis—(3′–5′)—cyclic dimeric GMP (cdGMP) (Zogaj et al., 2012). Overproduction of cdGMP in *F. novicida* U112 initiates biofilm formation as well as attenuates its ability replicate within mouse macrophages. The absence of these genes in *F. tularensis* suggests their elimination provided a selective advantage to its pathogenic intracellular life-cycle (Zogaj et al., 2012).

Gene amplification is evidenced in *F. tularensis* genomes as compared to *F. novicida* genomes. Most notably, genomic analyses of *F. tularensis* (6 subsp. tularensis, 12 subsp. holarctica, and 2 subsp. mediasiatica strains) and *F. novicida* (9 strains) indicate a duplication of the 30 kbp Francisella Pathogenicity Island (FPI) in *F. tularensis* as compared to *F. novicida* which contains only a single copy (Nano et al., 2004; Larsson et al., 2005, 2009; Rohmer et al., 2007). The FPI consists of 16–19 genes comprising a Type VI secretion system (T6SS) (Nano et al., 2004; Nano and Schmerk, 2007; De Bruin et al., 2011). Deletion of most genes within the FPI of both *F. tularensis* and *F. novicida* generates mutants that are defective for intra-macrophage growth and severely attenuated for virulence in mice (Tempel et al., 2006; Maier et al., 2007; Nano and Schmerk, 2007; Bröms et al., 2010; De Bruin et al., 2011; Chou et al., 2013). Given the importance of the FPI for intracellular replication and virulence, it seems likely duplication in *F. tularensis* represents a unique adaptation to its intracellular niche.

Only 7 genes unique to *F. tularensis* were identified via comparative genomic analysis of 20 *F. tularensis* strains (6 subsp. *tularensis*, 12 subsp. *holarctica*, and 2 subsp. *mediasiatica*); counterparts to these genes are absent in 9 *F. novicida* strains (Sjödin et al., 2012). All 7 genes are predicted to encode components necessary for the outer surface of *F. tularensis* cells (Sjödin et al., 2012). FTT0794, FTT0795, and FTT0796 are part of 12.5 kb locus important for formation of a capsule-like complex on the surface of *F. tularensis* (Bandara et al., 2011; Zarrella et al., 2011). The proteins encoded by these genes contain conserved domains for methyltransferase (FTT0795) and phosphocholine metabolism (FTT0794 and FTT0796) (Thomas et al., 2011). FTT1453c (wzx), FTT1454c (wbtJ), and FTT1458 (wzy) encode proteins involved in lipopolysaccharide O-antigen synthesis (Sjödin et al., 2012). The wbtJ gene of *F. tularensis* encodes an N-formyltransferase which converts the O-antigen sugar, dTDP-4,6-dideoxy-4-amino-D-glucose to dTDP-4,6-dideoxy-4-formamido-D—glucose, while the wzy gene product is an O-antigen polymerase whose function is to catalyze addition of newly synthesized O-antigen repeat units (Kim et al., 2010; Zimmer et al., 2013). FTT1188 encodes a hypothetical membrane protein lacking significant homology to known proteins (Sjödin et al., 2012).

Genomic analyses indicate that *F. tularensis* and *F. novicida* evolved as two distinct populations (Larsson et al., 2009). *F. tularensis* strains are highly clonal, differentiating them from *F. novicida* strains, which are characterized by a propensity for recombination. Recombination was noted in 10% of the 742 *Francisella* core genes tested in seven *F. novicida* genomes, whereas there was no evidence of recombination in these same genes when 20 *F. tularensis* genomes were examined (Larsson et al., 2009; Sjödin et al., 2012). Additionally, the *F. tularensis* Schu S4 genome shows evidence of 79 IS element insertions compared to only 26 IS element insertions in the *F. novicida* U112 genome (Rohmer et al., 2007). Genome decay due to IS element proliferation is clear in *F. tularensis;* IS elements in *F. tularensis* are responsible for at least 22 percent of inactivated genes (Larsson et al., 2009). IS element proliferation in *F. tularensis* is also proposed to be responsible for duplication of the FPI in *F. tularensis* (Rohmer et al., 2007; Larsson et al., 2009). Between *F. novicida* and *F. tularensis*, substantial differences are also observed in the ratio of substitution rates at non-synonymous and synonymous sites (dN/dS), with high dN/dS ratios for all *F. tularensis* branches, and considerably lower ratios for *F. novicida* (Larsson et al., 2009). Overall, these findings are consistent with the idea that niche restricted bacteria, such as intracellular pathogens, tend to have monomorphic genomes, whereas environmental bacteria are under weaker purifying selection and therefore retain the capacity to adapt to differing conditions by undergoing genomic changes (Moran, 2002; Achtman, 2008; Larsson et al., 2009).

## Virulence

The differing virulence between *F. tularensis* subspecies was classically determined by measuring the number of organisms required to kill 50–100% of infected mice, guinea pigs, and rabbits (Francis and Felton, 1953; Bell et al., 1955; Olsufiev et al., 1959). Variation in the time-to-death of *F. tularensis*-infected animals was also linked to virulence differences between *F. tularensis* subspecies (Olsufiev et al., 1959). Summarized in this section are results of virulence testing for *F. novicida* and *F. tularensis* by two routes of infection (subcutaneous and pulmonary) in mice, guinea pigs, rabbits, and rats. We note that the intent of this section is not to discuss the merits of using one animal model over another for tularemia research.

Both mice and guinea pigs are highly susceptible to *F. tularensis* (both subsp. *tularensis* and subsp. *holarctica*) when introduced via routes that mimic infection due to arthropod bite, with an observed LD_100_ of only 1 organism for subcutaneous inoculation (Bell et al., 1955; Olsufiev et al., 1959). The differing virulence between *F. tularensis* subsp. *tularensis* and *F. tularensis* subsp. *holarctica* in guinea pigs and mice manifests as a shortened time to death; *F. tularensis* subsp. *tularensis* infected mice and guinea pigs (<1000 organisms) die markedly earlier as compared to those infected with *F. tularensis* subsp. *holarctica* (Bell et al., 1955; Olsufiev et al., 1959). Differences in time to death of infected mice are also detected between subpopulations of *F. tularensis* subsp. *tularensis*. Intradermal infection of C57BL/6 mice with 10–20 CFUs results in significantly shortened survival times for those mice infected with A1b strains as compared to those infected with either A1a or A2 strains (Molins et al., 2010), consistent with human epidemiologic data indicating A1b strains have higher virulence than other *F. tularensis* subsp. *tularensis* strains (Kugeler et al., 2009).

In contrast to mice and guinea pigs, virulence is markedly different in rabbits between *F. tularensis* subsp. *tularensis* and *F. tularensis* subsp. *holarctica*. When introduced subcutaneously, an LD_100_ of 1 organism is observed for *F. tularensis* subsp. *tularensis* opposed to 10^9^ organisms for *F. tularensis* subsp. *holarctica* (Bell et al., 1955; Olsufiev et al., 1959). White rats are less susceptible to *F. tularensis* subsp. *tularensis* infection as compared to rabbits; an LD_100_ of 10^8^–10^9^ was reported for subcutaneous infection by either *F. tularensis* subsp. *tularensis* or *F. tularensis* subsp. *holarctica* (Olsufiev et al., 1959).

The virulence of *F. novicida* upon subcutaneous introduction appears to be less than *F. tularensis* in mice, guinea pigs and rabbits, although the exact extent of the difference is difficult to quantify. There is limited data in the literature with respect to the number of *F. novicida* organisms required to kill animals as determined by LD_50_ or LD_100_ studies. Similarly, there is a lack of published data comparing time to death of animals infected with *F. tularensis* vs. *F. novicida*. Initial experiments performed with *F. novicida* U112 indicated 50 organisms introduced subcutaneously was sufficient to kill 100% (4 of 4) of infected mice and guinea pigs (Larson et al., 1955). Owen et al. subsequently reported that 10–100 cells of *F. novicida* U112 were required to kill a mouse and 10–1000 cells required to kill a guinea pig, although no primary data or route of infection was provided (Owen et al., 1964). Experiments using BALB/cByJ mice infected intradermally with *F. novicida* U112 determined an LD_50_ of 2400 CFU (Kieffer et al., 2003). Much higher lethal doses for *F. novicida* introduced subcutaneously were reported in a study published in the Russian literature (Meshcheriakova et al., 1995). An LD_50_ of 1.3 × 10^4^ organisms and LD_100_ of ~10^7^, >10^8^, >10^8^ organisms was determined upon subcutaneous infection of outbred mice with *F. novicida* U112, *F. novicida* F6168, and *F. novicida* D9876, respectively (Meshcheriakova et al., 1995). In the same study, an LD_100_ >10^5^ organisms was identified for all three *F. novicida* strains (U112, F6168, D9876) via subcutaneous infection of guinea pigs, and in rabbits no mortality was observed with 10^8^ organisms of each strain (Meshcheriakova et al., 1995).

In recent years, the intranasal route of infection has been used to induce respiratory illness in mice, given the severity of pneumonic tularemia and the potential impact of an intentional aerosol release of *F. tularensis*. Published studies are consistent with a difference in virulence between *F. tularensis* subsp. *tularensis* Schu S4 and *F. novicida* U112 via this route of infection. An LD_50_ of <10 CFUs was determined for *F. tularensis* subsp. *tularensis* Schu S4 in BALB/c mice (Pechous et al., 2008) and 100% mortality is reported for infection of C57BL/6, BALB/c, and BALB/cByJ with 13-25 CFU (Qin et al., 2008; Cong et al., 2009; Child et al., 2010; Okan et al., 2013; Richard et al., 2014). For *F. novicida* U112, an approximate LD_50_ of 10 CFU was determined in inbred mice by intranasal inoculation, with two of five BALB/c mice surviving an inoculum of 30 CFU, and one of five surviving an inoculum of 300 CFU (Lauriano et al., 2004). In C57BL/6 and BALB/c mice, 100% mortality is reported using doses ranging from 100 to 445 CFU of *F. novicida* U112 (Pammit et al., 2004; Mares et al., 2008; Sharma et al., 2009).

Significant virulence differences between *F. tularensis* and *F. novicida* are evident upon pulmonary infection of Fischer 344 rats via intratracheal instillation (Ray et al., 2010). Fischer 344 rats show the highest sensitivity to *F. tularensis* subsp. *tularensis* Schu S4 (approximate LD_50_ of 5 × 10^2^ CFU) as compared to *F. tularensis* subsp. *holarctica* OR96-0246 (approximate LD_50_ of 1 × 10^5^ CFU) (Ray et al., 2010). In contrast, Fischer 344 rats are highly resistant to *F. novicida* U112 infection, with an approximate LD_50_ of 5 × 10^6^ CFU (Ray et al., 2010). Of note, a rapid time to death (MTD = 3 days) was observed in the rats which succumbed to infection with *F. novicida*, as compared to rats which died due to infection with *F. tularensis* (MTD = 10 days), suggesting death due to *F. novicida* was likely a toxic effect from the large number of organisms rather than from a productive infection (Ray et al., 2010).

## Mechanisms of pathogenesis

As described in this review, the genomes of *F. novicida* and *F. tularensis* are highly similar, with the vast majority of genes in *F. tularensis* also found in *F. novicida*. Despite this overall genetic similarity, evidence indicates differential regulation of and distinct roles for homologous genes in *F. tularensis* and *F. novicida* as pertains to pathogenesis. Moreover, *F. tularensis* has developed strategies distinct from *F. novicida* to evade host immune responses. This section will focus on some of the differences that have been described to date.

Evidence that the same genes in *F. novicida* and *F. tularensis* play distinct roles in pathogenesis comes from knockout studies of homologous genes. For example, inactivation of the genes encoding the transcriptional regulator IclR or the UDP-glucose-4-epimerase GalE resulted in attenuation of *F. novicida* U112, but not *F. tularensis* subsp. *tularensis* Schu S4 in a mouse model of infection (Weiss et al., 2007a; Mortensen et al., 2010; Thomas et al., 2011). In the case of the *dsbB* gene, which encodes disulfide bond formation B protein, deletion mutants were attenuated in both *F. tularensis* subsp. *tularensis* Schu S4 and *F. novicida* U112. However, *F. novicida* U112 knockouts provided protection from challenge with *F. novicida* U112, while *F. tularensis* subsp. *tularensis* Schu S4 mutants provided no homologous protection (Tempel et al., 2006; Qin et al., 2008). Intramacrophage secretion of FPI proteins also differs between *F. tularensis* and *F. novicida*. Upon infection of macrophages, 8 FPI proteins (IglE, IglC, IglI, IglJ, IglF, VgrF, PdpE, and PdpA) were secreted by *F. tularensis* subsp*. holarctica* LVS, whereas only 4 (IglE, IglC, PdpE, and PdpA) were secreted by *F. novicida*, suggesting fundamental differences may exist between the two species with respect to the Type VI secretion mechanism (Bröms et al., 2012).

The cell surface, a critical pathogenicity determinant, differs between *F. tularensis* and *F. novicida*. Early studies indicated a lack of serum cross-reactivity between *F. tularensis* and *F. novicida*. More recently all genes unique to *F. tularensis* as compared to *F. novicida* were predicted to encode outer surface components (see Genomics section) (Larson et al., 1955; Owen et al., 1964; Sjödin et al., 2012). Indeed, distinct structures for the core oligosaccharide and O-antigen of *F. tularensis* and *F. novicida* LPS have been described. The core oligosaccharide of *F. tularensis* lacks a glucose residue attached to the β-glucose branch as compared to *F. novicida* (Vinogradov et al., 2002; Vinogradov and Perry, 2004; Gunn and Ernst, 2007; Okan and Kasper, 2013), while the O-antigen of *F. tularensis* contains two distinct sugar moieties at either end of the tetra-saccharide repeat and is present in longer oligomer chains as compared to *F. novicida* (Vinogradov et al., 2004; Thomas et al., 2007; Barker et al., 2014). Reflecting the observed structural variation, three of the genes in the O-antigen encoding locus are unique to *F. tularensis* (see Genomics section) and among the other 12 genes, amino acid identity ranges from 98% to as low as 20% (Thomas et al., 2007; Sjödin et al., 2012). The structurally and antigentically unique O-antigens from *F. tularensis* and *F. novicida* appear to play different roles in the pathogenicity of each strain. In *F. tularensis*, the O-antigen is critical for intracellular survival as an O-antigen mutant (*wbtDEF*) (Thomas et al., 2007; Jones et al., 2012) is significantly attenuated for intracellular growth as compared to a similar *wbtDEF* mutant in *F. novicida*, which replicates normally in macrophages (Thomas et al., 2007). Recent evidence indicates that the O-antigen of *F. tularensis* subsp. *tularensis* Schu S4 protects it from autophagic detection once it reaches the cytosol (Case et al., 2014).

The cell surface of *F. tularensis* also plays an important role in cell entry and evasion of the host innate immune response. Within host serum, the function of complement proteins is to recognize pathogens and protect the host by direct lysis of the pathogen or opsonization leading to phagocytosis. Both *F. tularensis* and *F. novicida* have been shown to fix human complement protein C3 on their surface, but are resistant to complement mediated lysis due to rapid conversion of C3b to C3bi (Clay et al., 2008). This conversion of C3 leads to the interaction of C3bi with complement receptor protein C3R on host cells and cellular uptake by phagocytosis (Clemens et al., 2005; Ben Nasr and Klimpel, 2008; Clay et al., 2008; Dai et al., 2013). Deposition of C3 on *F. novicida* has been shown to increase both the production of reactive oxygen species (ROS) by human neutrophils and the production of TNFα, IL-6, and IL-1β by human monocytes (Barker et al., 2009; Dai et al., 2013). In stark contrast, C3 deposition on *F. tularensis* subsp. *holarctica* LVS resulted in significantly less ROS production by human neutrophils, and C3 deposition on *F. tularensis* subsp. *tularensis* Schu S4 was directly linked to suppression of the host immune response as monitored by the decreased production of the proinflammatory cytokines, TNFα, IL-6, and IL-1β, during uptake by human monocytes (Barker et al., 2009; Dai et al., 2013). Taken together, these results suggest different means of cellular entry for *F. tularensis* and *F. novicida* and also differential effects on the early host immune response.

A side-by-side comparison of pulmonary infection by *F. tularensis* or *F. novicida* in C57BL/6 mice demonstrated dissimilar cell types were infected *in vivo*. One day post-infection, via the intranasal route, *F. tularensis* subsp. *tularensis* Schu S4, *F. tularensis* subsp. *holarctica* LVS, and *F. novicida* U112 were preferentially associated with alveolar macrophages, although this proportion differed at 78.9, 70.3, and 51.6%, respectively (Hall et al., 2008). Strikingly, 27.3% of *F. novicida* infected cells on day 1 were neutrophils as compared to only 0 and 0.4% for *F. tularensis* subsp. *tularensis* Schu S4 and *F. tularensis* subsp. *holarctica* LVS, respectively (1000 fold difference in the number of neutrophils), indicating that neutrophils responded to and phagocytosed *F. novicida* U112 to a significantly greater extent than they did *F. tularensis* (Hall et al., 2008). Moreover, increasing numbers of alveolar macrophages and dendritic cells were infected from day 1 to 3 following inhalation with either *F. tularensis* subsp. *tularensis* Schu S4 or *F. tularensis* subsp. *holarctica* LVS, but not for *F. novicida* U112, suggesting more rapid killing of *F. novicida* infected cells (Hall et al., 2008).

Within host cells, *F. tularensis* and *F. novicida* display distinct abilities to evade the host immune response. The formation of the inflammasome, a multi-protein complex present in the host cell cytoplasm, is activated by microbial components to induce maturation of the inflammatory cytokines, interleukin IL-1β and IL-18, thereby leading to death of infected cells (Bauernfeind and Hornung, 2013). *F. novicida* is unable to efficiently evade this host innate immune response, and is recognized by the inflammasome upon escape from the phagosome and entry into the host cell cytoplasm (Mariathasan et al., 2006; Weiss et al., 2007b; Fernandes-Alnemri et al., 2010; Jones et al., 2012; Dotson et al., 2013). In contrast, *F. tularensis* successfully escapes inflammasome activation early in infection (~12 h) via a mechanism involving suppression of TLR2 signaling (Dotson et al., 2013). Presumably, this early suppression of the inflammasome allows *F. tularensis* time to successfully replicate to high levels in the cytoplasm prior to host cell death (Dotson et al., 2013).

Toll-like receptors (TLRs) play a central role in initiating innate cellular immune responses (Lim and Staudt, 2013). Evasion of TLR2 signaling has been shown to be involved in the intracellular replication of both *F. tularensis* and *F. novicida*, although the mechanism utilized diverges between the two bacteria (Telepnev et al., 2003; Katz et al., 2006; Malik et al., 2006; Abplanalp et al., 2009; Dai et al., 2013). Within the phagosome, *F. novicida* down-regulates the production of an endogenous transcript (FTN_1103), encoding a TLR2 stimulating lipoprotein, in a CRISPR/Cas system dependent manner (Sampson et al., 2013; Sampson and Weiss, 2013a,b). In contrast, *F. tularensis* lacks both the functional CRISPR/Cas system as well as the FTN_1103 homolog; genomic analyses indicate significant disruption/degradation of these genes (Schunder et al., 2013; Sampson and Weiss, 2013b). Rather, *F. tularensis* appears to evade TLR2 activation via a mechanism that involves the PI3K/Akt pathway, which when activated leads to production of the pro-inflammatory cytokines IL-6, IL-8, and IL-1β (Butchar et al., 2008; Cremer et al., 2009, 2011; Medina et al., 2010). The P13K/Act pathway is subject to negative regulation by the enzyme SHIP and a cellular micro-RNA, miR-155 (Cremer et al., 2009, 2011). Induction of miR-155 down-regulates SHIP to promote activation of the P13/Act pathway and inflammatory cytokine production. *F. tularensis* subverts or suppresses the induction of miR-155, thereby repressing the PI3K/Akt pathway. In contrast, *F. novicida* strongly induces miR-155, leading to activation of the P13K/Act pathway and the production of TNFα and IL-6 by human monocytes (Cremer et al., 2009, 2011).

## Treatment

Development of novel therapeutics for the treatment of tularemia is an area of active research given concern regarding the potential misuse of *F. tularensis* as a bioweapon. Standard antimicrobial therapy is effective for the treatment of tularemia, with aminoglycosides, tetracyclines, and chloramphenicol approved for treatment of tularemia by the U.S. Food and Drug Administration. Although ciprofloxacin and other fluoroquinolones are not currently FDA-approved for treatment of tularemia, they show very good efficacy against *F. tularensis in vitro*, in animals, and in humans (Johansson et al., 2000, 2002; Steward et al., 2006; Klimpel et al., 2008; Meric et al., 2008; Urich and Petersen, 2008; Nelson et al., 2010; Weber et al., 2012).

Antibiotic resistance to frontline therapeutics recommended for treatment of tularemia has never been identified in naturally occurring strains of *F. tularensis* or *F. novicida* (Ikäheimo et al., 2000; Garcia Del Blanco et al., 2004; Tomaso et al., 2005; Urich and Petersen, 2008; Valade et al., 2008; Georgi et al., 2012). Although treatment failure has been documented for human cases of tularemia, it is not associated with spontaneous antibiotic resistance, but rather a delay in antibiotic initiation (Celebi et al., 2006; Meric et al., 2008; Kaya et al., 2011). Nonetheless, antibiotic resistance remains a concern, whether spontaneous or intentionally engineered. *In vitro* experiments demonstrate that both *F. tularensis* and *F. novicida* have the ability to rapidly acquire resistance to quinolones. Passage of either *F. tularensis* subsp. *holarctica* LVS or *F. novicida* U112 on increasing concentrations of ciprofloxacin resulted in resistance to homologous classes of drugs (Sutera et al., 2014). Of note, in the case of *F. novicida*, but not *F. tularensis* subsp. *holarctica*, cross-resistance to heterologous classes of antimicrobials, including doxycycline and erythromycin, was observed (Sutera et al., 2014). This suggests *F. novicida* U112 encodes other genes not present in *F. tularensis* that confer multidrug resistance and is consistent with genomic comparisons indicating more transporters are present in the genome of *F. novicida* U112 (Rohmer et al., 2007; Sutera et al., 2014).

New therapeutic approaches for tularemia range from targeting the organism itself to modulating the host response in order to mount a protective response. These therapeutic approaches are covered in other chapters of this series. For approval and licensure of new therapeutics for tularemia, direct evaluation of the product's efficacy in a clinical setting is needed. Because therapeutic efficacy testing is not always feasible in a clinical setting (e.g., limited numbers of cases), the U.S. Food and Drug Administration developed the “Animal Rule” (21 CFR 314.610 and 21 CFR 601.91) to allow animal efficacy data to support product licensure or approval. Of note with respect to *F. tularensis* and *F. novicida*, the “Animal Rule” states that the etiological agent used in animal studies generally should be identical to the one that causes human disease. As discussed above, *F. novicida* does not cause tularemia in humans and differences between *F. novicida* and *F. tularensis* are also apparent in animals, indicating that *F. novicida* should not substitute for *F. tularensis* in efficacy testing of therapeutics. As recent studies indicate virulence differs among *F. tularensis* subsp. *tularensis* strains in humans (Kugeler et al., 2009; Molins et al., 2010), the use of more virulent A1b strains should be considered for therapeutic efficacy testing in animals.

## Conclusions and perspective

Bacterial species have traditionally been defined on the basis of DNA-DNA hybridization values (Lapage, 1992; Stackebrandt et al., 2002). The importance of phenotypic differences, however, cannot be understated with respect to classification of bacterial species. In 2002, the *ad-hoc* committee for the re-evaluation of bacterial species definition stated: “Phenotype, including chemotaxonomic markers, will remain important diagnostic properties in a species description. The ecological role can, in certain cases, decide on the species status. For example, medical organisms with defined clinical symptoms may continue to bear names that may not necessarily agree with their genomic relatedness so as to avoid unnecessary confusion among microbiologists and non-microbiologists [‘*nomen periculosum*’ according to Rule 56a(5) of the International Code of Nomenclature of Bacteria (Lapage, 1992)]” (Stackebrandt et al., 2002). A classic example of the value in utilizing phenotypic data to maintain distinct species designations comes from the bacteria *Yersinia pestis* and *Yersinia pseudotuberculosis*. Although these two bacteria share >97% nucleotide identity across 75% of their genes, they retain individual species names, due to their striking clinical and ecological differences (Chain et al., 2004; Carniel et al., 2006)*. Y. pestis* causes the highly fatal vector-borne disease, plague, whereas *Y. pseudotuberculosis* is transmitted by the fecal-oral route and infection rarely leads to death.

In this review, we have highlighted clinical, ecological, genomic, virulence, and pathogenic differences between *F. novicida* and *F. tularensis* that when considered in conjunction with genetic identity clearly warrants maintaining *F. novicida* and *F. tularensis* as separate species (Table 1)*. F. tularensis* causes the zoonotic vector-borne disease tularemia, whereas *F. novicida* does not. As determined by whole genome comparisons, *F. tularensis* evolved independently of *F. novicida*, which is consistent with its completely distinct ecological niche (*F. tularensis* is a zoonotic pathogen whereas *F. novicida* is not) and mechanisms of transmission (*F. tularensis* is transmitted by arthropod vectors whereas *F. novicida* is not). Moreover, as part of *F. tularensis'* pathogenic intracellular lifestyle, it has developed strategies distinct from *F. novicida* to evade host immune responses and successfully propagate in animal hosts.

*F. novicida* and its mutants have clearly contributed to our understanding of the biology of *F. tularensis*. A classic example was the discovery of the 30 kbp FPI in *F. novicida* (Gray et al., 2002; Nano et al., 2004). In more recent years, side-by-side experiments including both *F. novicida* and *F. tularensis* have highlighted the value of direct comparison between the two as pertains to understanding the unique pathogenic mechanisms *F. tularensis* has evolved to elicit its extreme virulence (Vinogradov et al., 2002, 2004; Vinogradov and Perry, 2004; Thomas et al., 2007; Butchar et al., 2008; Hall et al., 2008; Cremer et al., 2009; Mortensen et al., 2010; Bröms et al., 2012; Dai et al., 2013; Dotson et al., 2013; Sutera et al., 2014). As we move forward, findings utilizing the select agent exempt *F. novicida* U112 strain will no doubt continue to provide novel insight into the closely related species, *F. tularensis*. It is essential, however, to keep the two species separate and utilize standardized nomenclature for *F. novicida*. The recognition of *F. novicida* as a separate species via consistent and accepted nomenclature will limit misinterpretation of experimental results as pertains to the human disease tularemia caused by *F. tularensis*, avoid confusion between *F. tularensis* and *F. novicida* in clinical settings and ensure *F. tularensis* strains are used for treatment efficacy studies.

### Conflict of interest statement

The authors declare that the research was conducted in the absence of any commercial or financial relationships that could be construed as a potential conflict of interest.
